# Short- and mid-wavelength artificial light influences the flash signals of *Aquatica ficta* fireflies (Coleoptera: Lampyridae)

**DOI:** 10.1371/journal.pone.0191576

**Published:** 2018-02-07

**Authors:** Avalon Celeste Stevahn Owens, Victor Benno Meyer-Rochow, En-Cheng Yang

**Affiliations:** 1 Department of Biology, Tufts University, Medford, Massachusetts, United States of America; 2 Department of Genetics and Physiology, Oulu University, Oulu, Finland; 3 Research Institute of Luminous Organisms, Tokyo, Japan; 4 Department of Entomology, National Taiwan University, Taipei, Taiwan; Biocenter, Universität Würzburg, GERMANY

## Abstract

Urbanization can radically disrupt natural ecosystems through alteration of the sensory environment. Habitat disturbances are predicted to favor behaviorally flexible species capable of adapting to altered environments. When artificial light at night (ALAN) is introduced into urban areas, it has the potential to impede reproduction of local firefly populations by obscuring their bioluminescent courtship signals. Whether individual fireflies can brighten their signals to maintain visibility against an illuminated background remains unknown. In this study, we exposed male *Aquatica ficta* fireflies to diffused light of varying wavelength and intensity, and recorded their alarm flash signals. When exposed to wavelengths at or below 533 nm, males emitted brighter signals with decreased frequency. This is the first evidence of individual-level light signal plasticity in fireflies. In contrast, long wavelength ambient light (≥ 597 nm) did not affect signal morphology, likely because *A*. *ficta* cannot perceive these wavelengths. These results suggest long wavelength lighting is less likely to impact firefly courtship, and its use in place of broad spectrum white lighting could augment firefly conservation efforts. More generally, this study demonstrates benefits of bioluminescent signal plasticity in a “noisy” signaling environment, and sheds light on an important yet understudied consequence of urbanization.

## Introduction

As human populations grow, cities and suburbs expand into formerly natural lands [1]. Urbanization is a severe form of ecosystem disturbance, and one of the leading causes of species endangerment [2]. The impacts of deforestation, habitat fragmentation, and chemical pollution on species endemic to affected habitats have been widely recorded [3–6]. However, many species manage to survive and even thrive in urban centers, despite these myriad threats [7–10]. Opportunistic generalists such as cockroaches, rats, bats, and pigeons are inherently disposed to disturbed habitats [11], while other species persist through adaptation to their altered surroundings [12]. Population-level phenotypic change may occur over relatively long time scales, as has been observed in the case of industrial melanism in the peppered moth *Biston betularia* [13]. Concurrently, individual-level behavioral change can produce immediate fitness benefits. Urban populations of several bird species have been found to sing more loudly and at higher frequencies to compensate for increased ambient noise [14,15]. In these and other cases, signal plasticity promotes information transmission within noisy signaling environments [16].

In addition to noise, heat, and chemical pollution, urbanization commonly results in the introduction of artificial light at night (ALAN), the effects of which are infrequently studied in isolation [17]. ALAN transforms the nocturnal landscape: upward-directed “astronomical light pollution” obscures the night sky [18], while downward-directed “ecological light pollution” can affect species on the ground [17,19]. Among invertebrates, the attraction of moths and other flying insects to streetlamps is a particularly well studied phenomenon [20,21]. In contrast, the potential vulnerability of species with light-based communication systems to ALAN interference has only recently attracted the attention of researchers [22–28].

Bioluminescence has evolved multiple times within a broad range of bacterial, protist, fungal, and animal lineages, and is used in diverse contexts [29]. On land, bioluminescence is most common among insects: “glowworm” larvae of some fungus gnats use bioluminescent lures to attract prey [30], while four families of beetles, including the firefly family Lampyridae, have separately evolved a diverse range of light signals and signaling behavior [31,32]. Many adult fireflies use bioluminescent signals as part of courtship, with one or both sexes emitting prolonged glows, discrete flashes, or timed flash patterns to attract conspecifics [33–35]. During the mating season, adults begin flashing late in the day, when ambient light levels have declined to a species-specific threshold [36,37]. High levels of broad spectrum ALAN can delay or inhibit male signaling activity, and negatively affect female receptivity to bioluminescent signals [27,28].

How a particular light source impacts firefly activity depends in part on the intersection of its spectral emission with the spectral sensitivity of the species in question: if the firefly does not detect most wavelengths emitted by the source, courtship activity will likely be unaffected. Single copies of UV-sensitive (UVS) and long-wavelength-sensitive (LWS) opsin genes have been identified in multiple firefly species [38,39]. Peak sensitivity of the LWS visual pigment, after filtration by associated screening pigments, often corresponds to the peak wavelength of conspecific bioluminescence [40–43]. Short-wavelength-sensitive (SWS) opsins have yet to be found, although blue sensitivity has been described from electroretinograms (ERGs) and behavioral studies of multiple species [44–47]. The discrepancy may be due to opsin sensitization by as-yet-undescribed “antenna pigments” [48], or secondary interactions of screening pigments [42,45].

Recent studies have shown that broad spectrum white lighting negatively impacts firefly flash frequency (flashes/min) and/or the total number of flashing individuals present [27,28]. However, few studies have measured how specific wavelengths of ambient light interact with the firefly visual system and overlying neurobehavioral architecture to influence courtship behavior (but see [49,50] for studies of the spectral cues that initiate male flash activity). Different wavelengths are likely to have different effects. For example, receptive *Photinus pyralis* females emit response flashes exclusively to simulated male flashes of wavelengths longer than 480 nm, even though their eyes are sensitive to UV light [51]. To quantify the impact of different artificial light sources on firefly courtship behavior, we must compare the ways in which ambient light of different wavelengths affect firefly flash activity.

In this study we used monochromatic LEDs to test how specific wavelengths affect the signaling activity (intensity and frequency of flashes) of *Aquatica ficta* males. The resulting plot of activity vs. wavelength quantifies the effects of ambient light on two dimensions of *A*. *ficta* signal morphology, and positively identifies a range of wavelengths to which this species is visually sensitive. It is also the first controlled study of firefly signal plasticity, investigating the capacity for individual-level variation in flash intensity and frequency. Although likely too costly and inefficient under daytime illumination [52], some degree of signal plasticity may be used to promote signal visibility under twilight illumination, or in areas with low level ALAN. Thus we predict that *A*. *ficta* will flash more brightly under dim ambient light, but only of wavelengths to which they are visually responsive.

## Materials and methods

### Study organism

*A*. *ficta* is a common Taiwanese firefly notable both for its aquatic larvae and multivoltine life history [53]: most adults eclose in late April to May, but a second generation ecloses in mid-August [54]. *A*. *ficta* larvae require clean water and soft, mildly alkaline soil for development [54]. Conversion of streams into cemented irrigation ditches prevents these fireflies from using stream beds as oviposition or pupation sites, while water pollution and pesticide use may affect larvae and adults alike. The impacts of light pollution on *A*. *ficta* are unknown. Notably, small populations of *A*. *ficta* can still be found in a few areas within Taipei city center. Additionally, in 2016 the Taipei City Government Department of Public Works in collaboration with NGOs (re)introduced *A*. *ficta* to five prominent green spaces around Taipei: Da’an Forest Park, Muzha Cui Lake, Fuyang Eco Park, Zhongqiang Park, and Rongxing Garden [55].

Three male *A*. *ficta* fireflies were collected three times from Yongjian Eco Park (24.990195, 121.555824; May 4, May 7, and May 11, 2016), where they could be found approximately 20 min after sundown flying over a shaded stream emitting courtship flashes: periodic single yellow-green flashes (peak wavelength: 565 nm; bandwidth: 60 nm; n = 6) approximately 1 sec in duration. Specimens were transferred into transparent containers (250 ml volume) under natural conditions [54], and kept in an open-air courtyard by the National Taiwan University Insectarium. After a one day adjustment period, a single specimen was tested each evening over three consecutive evenings (no specimens were tested more than three days post-collection). Trials commenced 30 min after sunset and ran for 32 min, after which the specimen was released to prevent accidental retesting. The final sample size was limited by small local population numbers as well as the short adult lifespan and brief nightly activity period of this species.

### Artificial light

ALAN was simulated with eight LED pucks (Oznium.com, Pagosa Springs), circuit boards (diameter: 5 cm) populated by 30 monochromatic LEDs of identical peak wavelength, placed in plastic housing (diameter: 7 cm) and covered with 50% opaque frosted epoxy diffusers. Puck wavelengths were semi-evenly distributed across a range from near-UV to red (444 nm to 663 nm) in increments of 20–30 nm. Puck illumination was attenuated by a 72 mm H&Y adjustable neutral density filter (diameter: 7.5 cm, range: ND 0.3 to ND 2.4) before penetrating an opaque light chamber (S1 Fig). Ambient illumination intensity could be adjusted and observed in real time using a model 1935-C series power meter sensor (Newport) placed inside the light chamber. Trials were conducted in near darkness to minimize interference from outside light.

### Signal morphology

To obtain action spectra of firefly flash behavior, specimens were isolated within the light chamber, and the intensity over time of their light signals recorded and analyzed. Fireflies were secured to a piece of foam by a thin wire (22 ga) slid between their wings and dorsal abdomen. The act of restraint induced a series of fast (approx. 200 ms duration, 200–300 ms flash interval) alarm flashes in this species, which were produced with remarkable consistency throughout the entire experimental period. The foam was mounted to a slide cover, which was adjusted within a transparent acrylic slide cover box (2.5 cm^3^) to align the light organ of the specimen to the sensor of a USB4000 FLAME-S-XR1-ES spectrometer (Ocean Optics, Dunedin) connected to a Dell XPS 9343 laptop running SpectraSuite software (Windows Vista version 6.2). The “color chart” function of SpectraSuite was used to record average relative intensity (counts) of wavelengths from 565 nm to 569 nm over 32 min, capturing the peak wavelength of *A*. *ficta* bioluminescence. Averages were calculated and saved every 100 ms.

### Experimental protocol

After a 5 min adaptation period, each specimen underwent one 32 min trial without interruption, during which it was exposed to each of the eight pucks in a randomized order determined by a random sequence generator (http://www.random.org, S1 Table). For each wavelength, specimens were exposed to both dim (1×) and bright (10×) intensities; the order of exposure (i.e. dim or bright first) was secondarily randomized within wavelengths. Here intensity refers to quantum flux (photons per unit area per second), converted from power meter measurements of μW/cm^2^ and equalized across wavelengths: ranging from 0.107 ± 0.02 μmol m^-2^ s^-1^ and from 1.075 ± 0.212 μmol m^-2^ s^-1^ for dim and bright exposures, respectively. For both intensities of all wavelengths, exposures lasted 1 min and were always preceded by 1 min dark recordings, a total recording time of 4 min per wavelength.

### Data analysis

Eight sets of data were collected from eight *A*. *ficta* males on the evenings of May 5–9 and May 12–14, 2016 (S2 Fig; one male died before it could be tested). Of these, two recordings (May 5 and May 13) were disregarded due to measurement inconsistencies maintained over > 10 min of recording time. Flash intensity and frequency data from six individuals were separated into 1 min recordings (8×2×6 = 96 light recordings, and an equal number of dark recordings) and analyzed in R (version 3.2.3 [56]). The first 15 s of each recording was defined as the “adaptation period” (from light to dark or vice-versa) and omitted from subsequent analysis. Average intensity over time of light signals was zeroed within each recording to account for spectrometer noise, and average peak intensity calculated by isolating local maximums. Measurements ≤ 200 counts were excluded as noise, as peak intensity of *A*. *ficta* flashes consistently exceeded several thousand counts. The total number of peaks thus isolated was summed to obtain the average flash rate per individual for each exposure (peaks/min).

After removing the 15 s adaptation period, dark recordings still showed significant variation within and among individuals, suggesting high natural variation in *A*. *ficta* signal morphology as well as potential lingering effects of previous exposures (Fig 2A). Each set of light recordings was therefore compared to the initial set of dark recordings obtained previous to any ambient light exposure. Average flash intensity and frequency under each wavelength of exposure was compared using generalized linear mixed models (GLMM) from the R package ‘lme4’ [57]. Exposure wavelength was set as a fixed effect, and both specimen and exposure order (1 to 16; eight wavelengths × two intensities) initially included as random effects. Due to rank deficiency, both exposure intensities were analyzed separately, as were the effects on flash intensity and flash frequency (S1 Table). Exposure order explained a large amount of observed variation in flash frequency. However, it did not have an observable impact on flash intensity and was subsequently dropped from that set of models.

## Results

**Fig 1:**
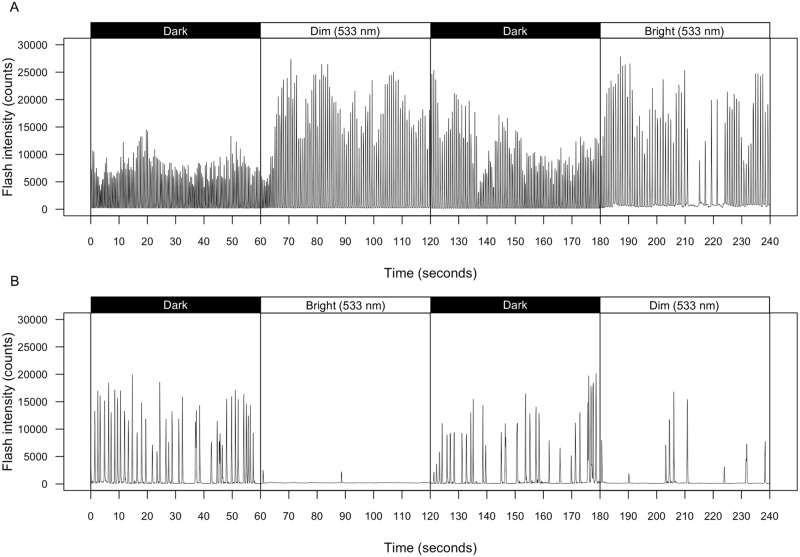
Plastic effects of 533 nm ambient light exposure on firefly flash morphology. (A) Example of change in intensity of flashes emitted by *A*. *ficta* male under different illumination conditions (indicated above each section), recorded in units of average counts per 100 ms over 4 min. (B) Changes in signal intensity over 4 min of separate *A*. *ficta* male under the same illumination conditions (indicated above each section).

**Fig 2:**
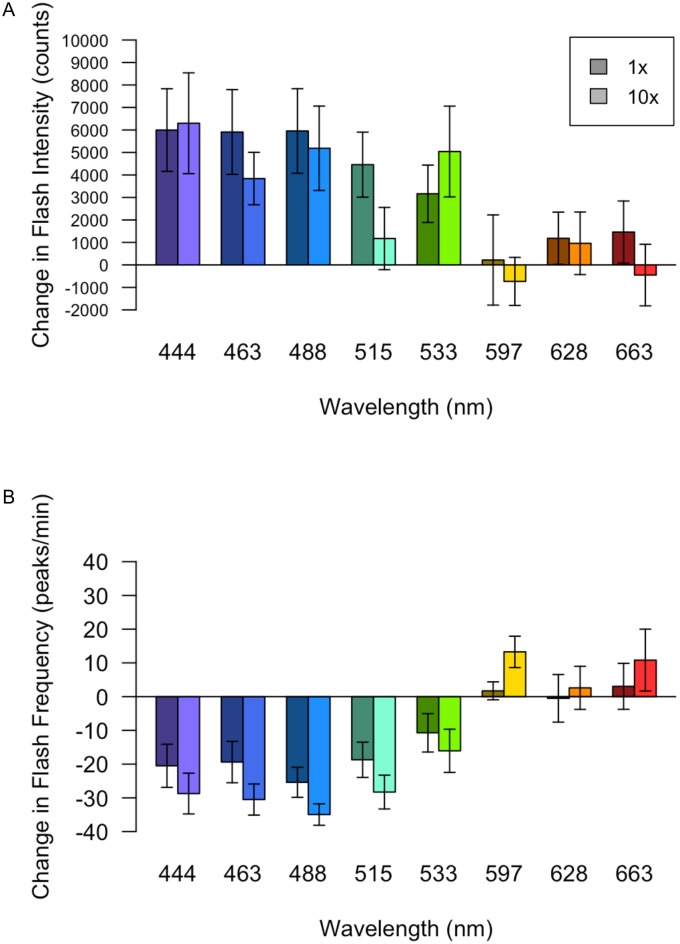
Effects of exposure wavelength and intensity on firefly flash morphology. Change in average (± 1 SE) flash intensity (A) and frequency (B) of light signals emitted by *A*. *ficta* under illumination by eight wavelengths and two intensities of ambient light (N = 6). Base values for average flash intensity (5420 counts) and flash frequency (43.167 peaks/min) obtained from 1 min dark recordings have been subtracted out.

*A*. *ficta* bioluminescent signaling behavior changed in response to environmental light (Fig 1A, Table 1). In comparison to base level data taken in complete darkness, average peak intensity of flash signals significantly increased, sometimes more than 100%, when individuals were exposed to dim intensities of short- and mid-wavelength light (Fig 2A, Table 1; 444 nm: p = 0.0021; 463 nm: p = 0.01; 488 nm: p = 0.017; 515 nm: p = 0.021; 533 nm: p = 0.066). Increases in flash intensity under dim short- and mid-wavelength illumination were accompanied by significant reductions in flash frequency (Fig 2B, Table 1; 444 nm: p = 0.0042, 463 nm: p = 0.049, 488 nm: p = p < 0.0001, 515 nm: p = 0.0098; 533 nm: p = 0.046). The decline is due to increases in interpulse interval [58] and, to a lesser extent, flash duration (S2 Table); in addition, sporadic periods without flashing were more common under illumination, sometimes continuing for 10 or more seconds. More extreme reductions in flash frequency were seen when males were exposed to bright intensities of the same wavelengths (Fig 2B, Table 1; 444 nm: p = 0.0019; 463 nm: p < 0.0001, 488 nm: p < 0.0001, 515 nm: p = 0.0018, 533 nm: p = 0.032). In fact, under bright short- and mid-wavelength illumination, three of six males ceased signaling entirely (Fig 1B). Due to the consequent lack of data, average peak intensity of flash signals became erratic under bright short- and mid-wavelength illumination (444 nm: p = 0.23; 463 nm: p = 0.19; 488 nm: p = 0.13; 515 nm: p = 0.59; 533 nm: p = 0.051). Notably, neither dim nor bright intensities of long wavelength (≥ 597 nm) illumination had a significant effect on flash intensity or frequency (p ≥ 0.3 in all instances; Fig 2).

**Table 1:** Average peak intensity and frequency of light signals produced by *A*. *ficta* under exposure to varying wavelengths and intensities of ambient light.

**Dim (1×) Exposure**						
**λ** (nm)	**flash intensity** $\bar{\text{x}}$ **± SD** (counts)	**degree of change relative to dark** (bright/dark)	**p-value**	**flash frequency** $\bar{\text{x}}$ **± SD** (peaks/min)	**degree of change relative to dark** (bright/dark)	**p-value**
444	11420 ± 4498	2.106	**0.0021**	22.67 ± 15.65	0.525	**0.0042**
463	11330 ± 4615	2.090	**0.010**	23.78 ± 15.02	0.551	**0.0049**
488	11380 ± 4605	2.099	**0.018**	17.78 ± 10.91	0.412	**0.000014**
515	9879 ± 3540	1.823	**0.021**	24.44 ± 12.81	0.566	**0.0098**
533	8584 ± 3125	1.584	0.066	32.44 ± 13.93	0.752	**0.046**
597	5639 ± 4912	1.040	0.94	44.89 ± 6.504	1.040	0.30
628	6605 ± 2848	1.219	0.91	42.67 ± 17.28	0.988	0.55
663	6881 ± 3381	1.269	0.72	46.22 ± 16.67	1.071	0.43
**Bright (10×) Exposure**						
**λ** (nm)	**flash intensity** $\bar{\text{x}}$ **± SD** (counts)	**degree of change relative to dark** (bright/dark)	**p-value**	**flash frequency** $\bar{\text{x}}$ **± SD** (peaks/min)	**degree of change relative to dark** (bright/dark)	**p-value**
444	11719 ± 5489	2.162	0.23	14.44 ± 14.80	0.335	**0.0019**
463	9259 ± 2854	1.708	0.19	12.67 ± 11.29	0.293	**0.00042**
488	10606 ± 4595	1.957	0.13	8.222 ± 7.78	0.190	**0.000014**
515	6594 ± 3388	1.217	0.59	14.89 ± 12.29	0.345	**0.0018**
533	10462 ± 4941	1.930	0.051	27.11 ± 15.70	0.628	**0.032**
597	4688 ± 2615	0.865	0.48	56.44 ± 11.38	1.308	0.92
628	6381 ± 3407	1.177	0.97	45.78 ± 15.61	1.060	0.56
663	4968 ± 3351	0.917	0.98	54.00 ± 22.45	1.251	0.87

1× dim exposures (top) and 10× bright exposures (bottom) differ by one order of magnitude. Change from dark for each exposure is calculated by dividing by base values taken from 1 min dark recordings (average flash intensity: 5420 counts; frequency: 43.167 peaks/min).

Although some males underwent remarkably rapid changes in average flash intensity, individuals often appeared to remain at least partially affected by the previous series of exposures (Fig 1A and 1B). When analysis of variance (ANOVA) was used to compare models, results showed that number of previous exposures had a significant negative effect on flash frequency (1×: p < 0.0001, χ^2^ = 15.205; 10×: p = 0.0012, χ^2^ = 14.668), but not flash intensity (p = 1.0 and χ^2^ = 0 in both 1× and 10× models). Thus, the brightness of male flashes was apparently only influenced by the current level of ambient illumination, while the total number of flashes per minute was determined by both current and previous light exposures.

## Discussion

Our results suggest that *A*. *ficta* is not visually responsive to red wavelengths. However, short wavelength ambient light (including blue) significantly affects their signaling behavior, inducing increased flash intensity as well as decreased flash frequency. Erratic measurements obtained during bright (10×) exposures across wavelengths may reflect the small sample size, necessitated by the limited population numbers and highly seasonal life history of *A*. *ficta*, or conflict between two opposing behaviors: 1.) increasing light signal intensity in response to low level environmental light and 2.) ending light signal production in response to high level environmental light. The latter behavior likely relates to entrainment of diurnal and nocturnal modes of activity: fireflies do not engage in courtship activity during their subjective daytime [36,37]. The former may be an adjustment to the nocturnal mode of activity, a plastic response to increases in environmental light levels within some “nighttime” range.

This is the first description of individual-level variation in the average peak intensity of firefly flash signals. Previous studies on the characteristic flash patterns of North American *Photinus* fireflies have described intraspecific variation in several other signal parameters. These include flash rate, flash duration, and interpulse interval [59–62], in addition to flash frequency (total flashes/min) [27,28,36,59] and flash wavelength [63]. Individual- and/or population-level variation has been shown to be related to age [64], habitat type [63], temperature, humidity, time of day, and ambient light [27,28,36]. Our results open up another dimension of signal morphology for future behavioral studies. They also suggest that while fireflies can brighten their flashes in response to ambient light, this potentially beneficial behavior may require some decrease in average flash frequency [correlation coefficient: -0.9655009 (1×), -0.8027756 (10×)].

The plastic increases in alarm flash intensity we observed during dim (1×) exposures may be a product of natural or sexual selection. Alarm flashes, which serve as aposematic signals [65,66], will be more easily perceived by potential predators if they have greater contrast against the background. When given a choice among simulated courtship flashes of varying intensity, *P*. *pyralis* males and females [67,68] and *L*. *noctiluca* males [46] all prefer brighter signals, likely due to their greater visibility. Temporary increases in signal intensity could also help to maintain a base level of visibility against artificially illuminated backgrounds. Resilience of urban firefly populations to low level ALAN will depend on the success with which these signal modifications maintain inter- and intraspecific lines of communication, and the fitness costs of doing so. Flash signal production may have a relatively low metabolic cost [69]. If this is the case, increasing the average intensity of one’s light signals could help ensure continued predator deterrence and/or courtship success without any corresponding fitness cost, offering a fascinating case study of the benefits of behavioral plasticity in urbanized environments. However, other evidence suggests that flash production does require high levels of lipid metabolism [70], and therefore certain tradeoffs (such as decreased flash frequency) may be involved.

Regardless of metabolic cost, even extremely bright flashes should be impossible to perceive in sufficiently bright environments, which may be why half of the *A*. *ficta* specimens stopped flashing under bright (10×) exposures (1× and 10× exposures roughly correspond to 20 and 200 lux, respectively, although photometric units are not suited to the analysis of monochromatic light; see S1 Text). Fireflies do not flash under daytime levels of artificial or natural illumination [36,37]. Hagen *et al*. [27] observed significant decreases in the average number of flashing *Photinus* sp1 individuals encountered in brightly lit conditions (1.5–4.45 lux). Firebaugh and Haynes [28] also found decreases in flash frequency (flashes/min, a proxy for abundance) of *Photuris versicolor* in an experimental plot lit by LED floodlights to ~301 lux. However, flash frequency of *Photinus pyralis* males was unaffected. *P*. *pyralis* is a common crepuscular species, while *P*. *versicolor* is nocturnal and may be less resilient to changes in the light environment. Despite this, the receptivity of *P*. *pyralis* females to male signals decreased in experimental plots. Females may have been unable to see these signals, or less receptive to them due to their decreased contrast against the background; females are likely unable to distinguish decreases in contrast (perceived intensity) from decreases in emitted intensity. Plastic increases in male flash intensity are unlikely to greatly promote visibility in this context, due to the extreme brightness of the artificial light source.

Selecting artificial light sources that have the least chance of interfering with firefly communication can help to reduce disturbance of urban firefly populations. Blue light is often employed in firefly observations and experiments in the west, as it is thought to be minimally disruptive to local species. Although thus far genetic analysis has failed to uncover blue opsin genes in any firefly species [38,39], we found that blue wavelengths did significantly affect the flash signaling behavior of *A*. *ficta*. Our results suggest artificial light sources emitting the majority of their light in wavelengths ≥ 597 nm, including a range of yellow to red wavelengths visible to humans, would be most suitable for *A*. *ficta* habitats. These conclusions receive support from ERG spectral sensitivity studies of *Luciola cruciata*, a close relative of *A*. *ficta*, which have described sensitivity peaks in the green-yellow range of 555–565 nm wavelengths [71,43]. Low-pressure sodium vapor lamps emit light concentrated at 590 nm, well beyond this range [72]. However, in recent decades these lamps have been phased out in favor of brighter and more energy efficient alternatives such as LEDs, which often emit a large portion of their light in the blue region of the spectrum. We suggest spectral emission be given greater consideration when installing artificial lights around firefly habitats. The directionality of light sources (streetlamps *vs*. path lights) and distinction between point sources and larger sources of diffused light are also relevant to these discussions. Free-roaming *A*. *ficta* in the lab continue to emit courtship signals when exposed to small points of light, regardless of the intensity or distance of the point source from their eyes, although they appear to exhibit some degree of negative phototaxis (*pers*. *obs*.). However, *A*. *ficta* respond to high levels of short wavelength diffused light by decreasing the frequency of their flash signals, and eventually ceasing them altogether.

*A*. *ficta* do not exchange precisely timed flash patterns during courtship, so decreases in flash frequency like those we observed in dim light conditions may not cause a significant loss of signal information, and the commensurate increase in intensity may suffice to maintain signal visibility in natural settings. It remains to be seen how ambient light affects the courtship of *Photinus* fireflies, which use flash patterns to communicate information about species identity and individual fitness. Changes to flash rate, flash duration, or interpulse interval are highly likely to impact the reproductive success of these species; even slight differences in flash rate have been shown to affect male reproductive success [60,73].

Much remains to be done to quantify the total fitness impact of ALAN of varying spectral composition on firefly species of varying spectral sensitivity and courtship behavior. Observed decreases in *A*. *ficta* populations over the past few decades indicates that they have been negatively affected by habitat alterations occuring within this time (Wu Chiahsiung, personal communication). The impacts of habitat destruction, climate change, pesticides, and ALAN on fireflies are all relevant, and likely mutually reinforcing, but those of ALAN deserve equal consideration. This is especially the case because fireflies can serve as “flagship species”, charismatic mascots of public outreach efforts to reduce or eliminate ALAN in urban areas. Improving our understanding of the degree to which fireflies everywhere are able to respond and adapt to ALAN is crucial should we hope to protect these species going forward, both for the robustness of the ecosystem and the enjoyment of many generations to come.

## Supporting information

S1 Fig Simplified schematic of experimental setup. LED pucks fitted into upper box (base: 14×8.5 cm; height: 3 cm) shine through a neutral density filter, the transparency of which is adjusted via rotation of this box. Walls of the light chamber (base: 9×6.5 cm; height: 5.5 cm) are covered in opaque dark room fabric. The USB4000 spectrometer is connected to a Dell laptop running SpectraSuite software. Walls of the light chamber (base: 9×6.5 cm; height: 5.5 cm) are covered in opaque dark room fabric.
(TIF)

S2 Fig Signal morphology of eight *A*. *ficta* males over eight separate 32 min trials. During each trial, one unique individual was exposed to two intensities of eight wavelengths of LED for 1 min each. Trial date is given to the left of each recording. All light exposures were preceded by a 1 min dark exposure, summing to 32 exposures total (16 light and 16 dark). Exposure order (intensity and wavelength) was randomized; semi-transparent colored overlays indicate the series of exposure intensities and wavelengths for each recording, corresponding to the colors used in Fig 2 and S1 Table. Bioluminescence was recorded in units of average intensity (counts) per 100 ms.
(TIF)

S1 Table Sample experimental procedure, taken from trial on May 5, 2016. Trial began 30 min post-sunset at 18:56. The order of LED wavelengths has been randomized, as has the order of exposure intensity (dim or bright first) within wavelengths. Relative quantum flux density (μmol m^-2^ s^-1^) is approximately equal across wavelengths; variation in energy measurements (μW/cm^2^) reflects inherent differences in the energy of photons of different wavelength.
(DOCX)

S2 Table Change in *A*. *ficta* flash duration and interpulse interval under short- and mid-wavelength illumination. Data from four of eight experimental trials, organized by trial date, are given above. The first column contains average values of flash duration and interpulse interval (duration between flashes) from the initial 1 min dark recording of each insect. The following columns contain average values for all five short- to mid-wavelength exposures (444–533 nm), at 1× and 10× intensity, respectively.
(DOCX)

S1 Text Relevance of experimental design to urban and suburban light environments. (DOCX)
