# Supplementary figures and images for: Short- and mid-wavelength artificial light influences the flash signals of Aquatica ficta fireflies (Coleoptera: Lampyridae)
Source: PLoS One. 2018 Feb 7;13(2):e0191576. doi: 10.1371/journal.pone.0191576 (PMC5802884; doi:10.1371/journal.pone.0191576)

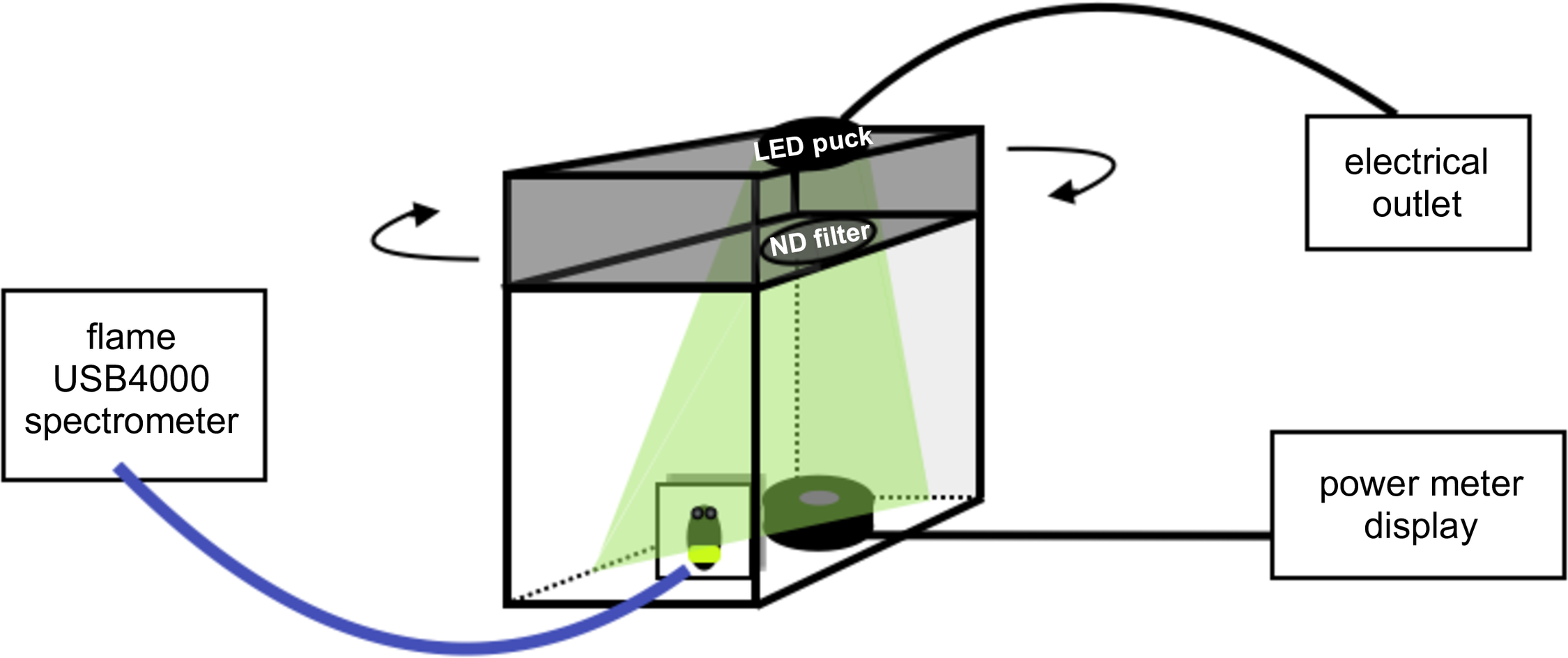

Supplement: S1 Fig — LED pucks fitted into upper box (base: 14×8.5 cm; height: 3 cm) shine through a neutral density filter, the transparency of which is adjusted via rotation of this box. Walls of the light chamber (base: 9×6.5 cm; height: 5.5 cm) are covered in opaque dark room fabric. The USB4000 spectrometer is connected to a Dell laptop running SpectraSuite software. Walls of the light chamber (base: 9×6.5 cm; height: 5.5 cm) are covered in opaque dark room fabric. (TIF) [file pone.0191576.s001.tif]

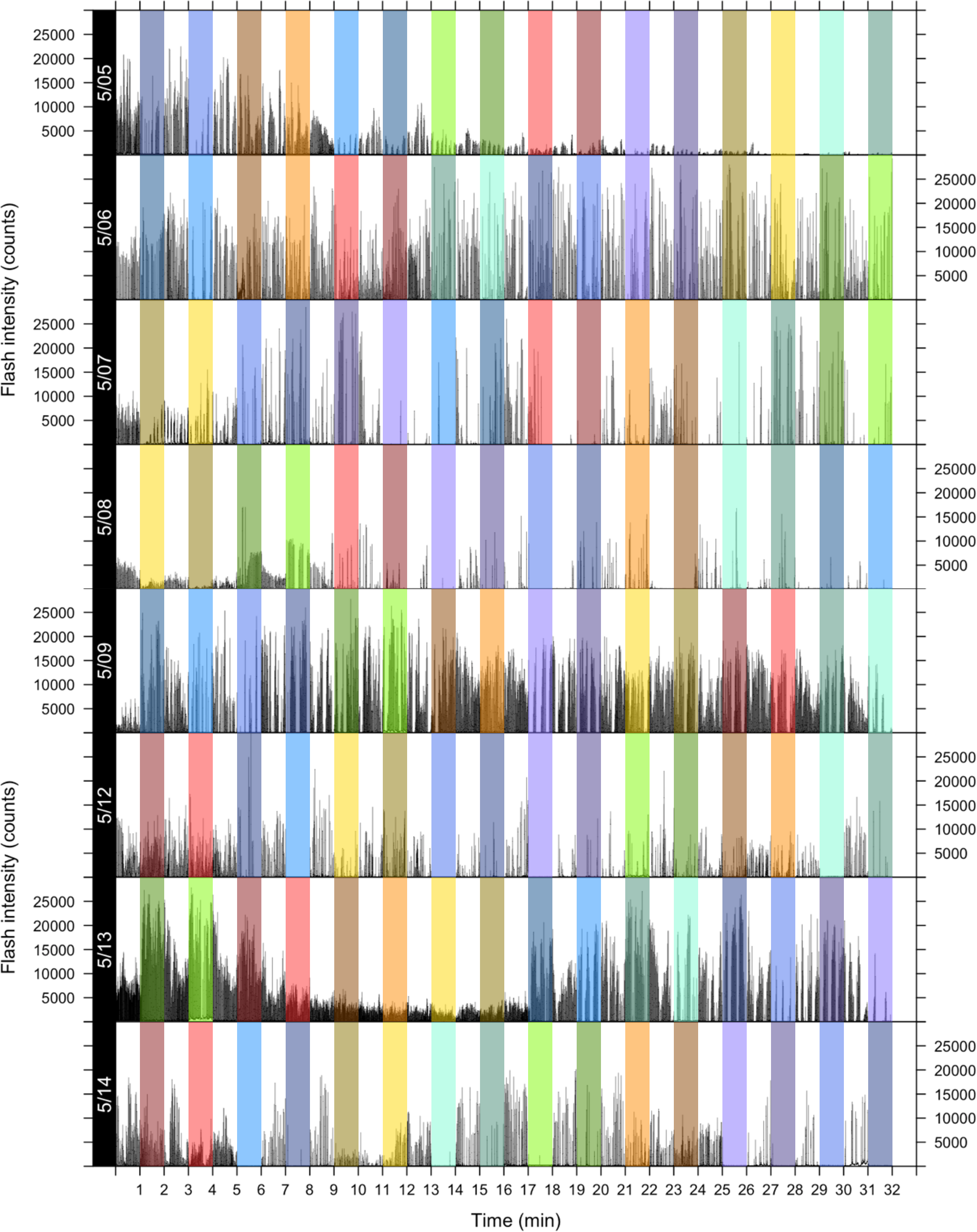

Supplement: S2 Fig — During each trial, one unique individual was exposed to two intensities of eight wavelengths of LED for 1 min each. Trial date is given to the left of each recording. All light exposures were preceded by a 1 min dark exposure, summing to 32 exposures total (16 light and 16 dark). Exposure order (intensity and wavelength) was randomized; semi-transparent colored overlays indicate the series of exposure intensities and wavelengths for each recording, corresponding to the colors used in Fig 2 and S1 Table. Bioluminescence was recorded in units of average intensity (counts) per 100 ms. (TIF) [file pone.0191576.s002.tif]
