# Supplementary material for: Short- and mid-wavelength artificial light influences the flash signals of Aquatica ficta fireflies (Coleoptera: Lampyridae)
Source: PLoS One. 2018 Feb 7;13(2):e0191576. doi: 10.1371/journal.pone.0191576 (PMC5802884; doi:10.1371/journal.pone.0191576)
Supplement: S1 Table — Trial began 30 min post-sunset at 18:56. The order of LED wavelengths has been randomized, as has the order of exposure intensity (dim or bright first) within wavelengths. Relative quantum flux density (μmol m-2 s-1) is approximately equal across wavelengths; variation in energy measurements (μW/cm2) reflects inherent differences in the energy of photons of different wavelength. (DOCX) [file pone.0191576.s003.docx]

**S1 Table.** **Sample experimental procedure, taken from trial on May 5, 2016.**

| **May 5th** | | | | | | | | | | | | | | | | |
| --- | --- | --- | --- | --- | --- | --- | --- | --- | --- | --- | --- | --- | --- | --- | --- | --- |
| **λ (nm)** | **463** | | | | **628** | | | | **488** | | | | **533** | | | |
| **intensity**  **(μW/cm^2^)** | **0** | **3.7** | **0** | **37** | **0** | **27** | **0** | **2.7** | **0** | **35** | **0** | **3.5** | **0** | **32** | **0** | **3.2** |
| **time**  **(MM:SS)** | *00:01 - 04:00* | | | | *04:01 - 08:00* | | | | *08:01 - 12:00* | | | | *12:01 - 16:00* | | | |
| **λ (nm)** | **663** | | | | **444** | | | | **597** | | | | **515** | | | |
| **intensity**  **(μW/cm^2^)** | **0** | **26** | **0** | **2.6** | **0** | **39** | **0** | **3.8** | **0** | **2.9** | **0** | **29** | **0** | **33** | **0** | **3.3** |
| **time**  **(MM:SS)** | *16:01 - 20:00* | | | | *20:01 - 24:00* | | | | *24:01 - 28:00* | | | | *28:01 - 32:00* | | | |

Trial began 30 min post-sunset at 18:56. The order of LED wavelengths has been randomized, as has the order of exposure intensity (dim or bright first) within wavelengths. Relative quantum flux (µmol m^-2^ s^-1^) is approximately equal across wavelengths. Variation in energy measurements (μW/cm^2^) reflects the varying energy of photons of different wavelength.
