# Supplementary material for: Short- and mid-wavelength artificial light influences the flash signals of Aquatica ficta fireflies (Coleoptera: Lampyridae)
Source: PLoS One. 2018 Feb 7;13(2):e0191576. doi: 10.1371/journal.pone.0191576 (PMC5802884; doi:10.1371/journal.pone.0191576)
Supplement: S2 Table — Data from four of eight experimental trials, organized by trial date, are given above. The first column contains average values of flash duration and interpulse interval (duration between flashes) from the initial 1 min dark recording of each insect. The following columns contain average values for all five short- to mid-wavelength exposures (444–533 nm), at 1× and 10× intensity, respectively. (DOCX) [file pone.0191576.s004.docx]

| **May 6th** | | | |
| --- | --- | --- | --- |
|  | Dark | Dim | Bright |
| Flash Duration (sec) | 2.607237 | 3.050113 | 2.918822 |
| Interpulse Interval (sec) | 11.571054 | 17.77855 | 19.220221 |
| **May 7th** | | | |
|  | Dark | Dim | Bright |
| Flash Duration (sec) | 2.325351 | 3.376282 | 3.091775 |
| Interpulse Interval (sec) | 51.433639 | 69.180128 | 52.881212 |
| **May 9th** | | | |
|  | Dark | Dim | Bright |
| Flash Duration (sec) | 2.299149 | 2.931343 | 2.848132 |
| Interpulse Interval (sec) | 6.643455 | 12.763707 | 12.975615 |
| **May 13th** | | | |
|  | Dark | Dim | Bright |
| Flash Duration (sec) | 2.138064 | 2.657381 | 2.530629 |
| Interpulse Interval (sec) | 6.831846 | 7.984212 | 20.091429 |

**S2 Table. Change in *A. ficta* flash duration and interpulse interval under short- and mid-wavelength illumination.**

Data from four of eight experimental trials, organized by trial date, are given above. The first column contains average values of flash duration and interpulse interval (duration between flashes) from the initial 1 min dark recording of each insect. The following columns contain average values for all five short- to mid-wavelength exposures (444 – 533 nm), at 1× and 10× intensity, respectively.
