# Supplementary material for: Short- and mid-wavelength artificial light influences the flash signals of Aquatica ficta fireflies (Coleoptera: Lampyridae)
Source: PLoS One. 2018 Feb 7;13(2):e0191576. doi: 10.1371/journal.pone.0191576 (PMC5802884; doi:10.1371/journal.pone.0191576)
Supplement: S1 Text — (DOCX) [file pone.0191576.s005.docx]

**S1 Text. Relevance of experimental design to urban and suburban light environments.**

Assessments of the brightness of monochromatic light are complicated by the fact that standard photometric measures of brightness (lux, lumens, *etc.*) have been scaled to reflect human spectral sensitivity. Because of this, light from the LED pucks used in this experiment was of radically different photometric intensity, even though the radiometric intensity (photons/s) was the same across exposure type. Our highest lux measurements were obtained from 533 nm green LEDs, and we suggest using these figures to make comparisons: approximately 20 lux at 1× intensity and 200 lux at 10× intensity.

A night sky with a full moon is only 0.05 to 0.36 lux [1]. However, twilight on a moonless night ranges from approximately 1000 lux at the beginning of civil twilight to almost 0 lux at the end of astronomical twilight, and the final intensity is greater on a cloudy night in a light polluted area [2]. The intensity of ALAN sources such as street lamps and house lights depends on bulb type, wattage, and the distance of the source from the animal. While the NOAO recommended light level for public areas with dark surroundings is only 20 to 50 lux (www.noao.edu), LED street lamps can reach intensities of 80 lux or more (www.dmxledlights.com), depending on the distance of the illuminated surface (or insect) from the light.

Because fireflies are small-bodied insects, a bright street lamp overhead could radically alter ambient light in their “microhabitat,” similar to how ambient light was altered in our experiments. In light of these considerations, our data are relevant for fireflies in urban and suburban environments, and have interesting implications for crepuscular fireflies in rural environments as well.

1. Kyba C, Mohar A, Posch T. How bright is moonlight? A&G 2017;58: 1.31–1.32.

2. Cronin TW, Johnsen S, Marshall JM, Warrant EJ. Visual Ecology. Princeton University Press; 2014.
